# Which Biomass Stove(s) Capable of Reducing Household Air Pollution Are Available to the Poorest Communities Globally?

**DOI:** 10.3390/ijerph18179226

**Published:** 2021-09-01

**Authors:** Debbi Stanistreet, Eunice Phillip, Nitya Kumar, Rachel Anderson de Cuevas, Megan Davis, Jessica Langevin, Vincent Jumbe, Aisling Walsh, Sarah Jewitt, Mike Clifford

**Affiliations:** 1 Department of Public Health and Epidemiology, Royal College of Surgeons University of Medicine and Health Sciences, D02DH60 Dublin, Ireland; eunicephillip@rcsi.ie (E.P.); MeganDavis@rcsi.ie (M.D.); JessicaLangevin@rcsi.ie (J.L.); aislingwalsh@rcsi.ie (A.W.); 2 Department of Medicine, Royal College of Surgeons in Ireland University of Medicine and Health Sciences, Manama 15503, Bahrain; nkumar@rcsi-mub.com; 3 Department of Public Health, Policy and Systems University of Liverpool, Liverpool L69 3GF, UK; r.anderson-de-cuevas@liverpool.ac.uk; 4 Department of Health Systems and Policy, Kamuzu University of Health Sciences, Mahatma Gandhi, Blantyre 560001, Malawi; vjumbe@medcol.mw; 5 School of Geography, University of Nottingham, Nottingham NG7 2RD, UK; sarah.jewitt@nottingham.ac.uk; 6 Faculty of Engineering, University of Nottingham, Nottingham NG7 2RD, UK; Mike.Clifford@nottingham.ac.uk

**Keywords:** improved cookstoves, household air pollution, global poorest, SDG 7, clean fuel access

## Abstract

Globally, household and ambient air pollution (HAAP) leads to approximately seven million premature deaths per year. One of the main sources of household air pollution (HAP) is the traditional stove. So-called improved cookstoves (ICS) do not reduce emissions to levels that benefit health, but the poorest communities are unlikely to have access to cleaner cooking in the medium term. Therefore, ICS are being promoted as an intermediate step. This paper summarises the current evidence on the ICS available to the global poorest, utilising data from the Clean Cookstoves Catalog and systematic review evidence from the field. The cheapest stoves offer little reduction in HAP. Only one ICS, available at US$5 or less, (the canarumwe) minimally reduced pollutants based on ISO testing standards and no studies included in the systematic reviews reported tested this stove in the field. We recommend field testing all ICS as standard, and clear information on stove characteristics, sustainability, safety, emissions efficiency, in-field performance, affordability, availability in different settings, and the ability of the stove to meet community cooking needs. In addition, ICS should be promoted alongside a suite of measures, including improved ventilation and facilities to dry wood, to further reduce the pollutant levels.

## 1. Introduction

Each year, the combined effects of household and ambient air pollution (HAAP) lead to approximately seven million premature deaths globally [1]. Sources of HAAP in low- and middle-income countries include the incomplete combustion of charcoal and biomass in households using open fires and traditional cookstoves for cooking and also kerosene for lighting [2]. The effects of HAAP lead not only to direct ill-health effects from inhalation of toxic gases, such as carbon monoxide (CO), and fine particulate matter (PM_2.5_), but also to indirect effects due to environmental damage through deforestation and the presence of climate-depleting compounds, such as black carbon [3].

The implications of HAAP for health have been widely reported. HAAP causes pneumonia in children, cardiovascular and chronic obstructive pulmonary disease, and lung cancer in adults [4]. It is also associated with cataract development [5], adverse pregnancy outcomes [6], and according to the World Health Organization (WHO), approximately 265,000 deaths occur each year from fire-related injuries from traditional stoves [7]. Although the morbidity and mortality from HAAP is of global concern, the burden is highest in low- and middle-income countries, among the poorest populations, and in women and children living in housing with only a single room for shelter [8], contributing to the global widening of health inequalities. Further, women are overwhelmingly responsible for fuel collection and cooking, further increasing their health risks, but also limiting their economic opportunities [9].

Sustainable Development Goal (SDG) 7 focuses on promoting access to affordable, reliable, and sustainable energy services for all, which for most means access to gas and electricity, and is also crucial to the success of most of the other 16 SDGs. Despite its importance, in terms of progress, universal access by 2030 (Target 7.1) is highly unlikely to be achieved among the global poorest, including among approximately one quarter of the world’s population, living on less than US$3.20 a day [10]. Further, this number is expected to rise in the future due to the COVID-19 pandemic, with impacts on conflict and also climate change [11].

### 1.1. The Global Poorest Communities and Access to Clean Energy

The WHO Indoor Air Quality (IAQ) Guidelines [12] recommend that implementing agencies work to increase access to, and sustained use of, clean fuels as widely and rapidly as is feasible, and much vital work is ongoing in this respect in many parts of the world promoting LPG and electricity. However, according to the World Energy Outlook (WEO), while the share of people lacking access to electricity and clean cooking is expected to decline by 2030, the absolute numbers of those without access in Africa will increase [13]. Further, the WEO estimates that the number of people without access to clean cooking facilities will be 2.3 billion in 2030 and will only have fallen to 1.8 billion in 2040. The population without access to clean energy will be almost equally shared between Africa and developing Asia by 2040, affecting the health and wellbeing of the most vulnerable (women and children) in the poorest communities [13].

Given that global progress is falling far short of meeting this universal access target, it is crucial to begin a dialogue on what options are realistically available to the global poorest communities in Africa and Asia or they risk being left behind in the move towards clean energy. Further, the COVID-19 pandemic has caused more disruption to the energy sector than any other event in recent history with reports that access to cleaner household fuels has become variable and disrupted, which is likely to worsen the outlook in terms of progress to cleaner fuels [14]. This emphasises the importance of increasing access to clean and safe household energy ensuring the delivery of genuine health gains [15].

This paper reviews the available evidence on improved biomass stoves in relation to household air pollution (HAP) reduction using the widely accepted International Organisation for Standardisation (ISO) guidelines, and discusses the findings in relation to evidence from systematic reviews in the field, outlining the practical options available to support the global poorest communities to reduce their personal exposure to air pollution within the household.

### 1.2. Current Approaches Available to the Global Poorest to Reduce HAP in the Short to Medium Term

HAP results from a number of different sources, but the main focus of much research and scale-up programmes in the field in low-income countries has been on access to cleaner cooking. For the poorest communities who do not have access to gas and electricity, there are numerous clean stoves on the market in low-income settings, including solar, biogas, and ethanol stoves. However, evidence suggests that these stove options are unlikely to be the answer to large-scale HAP reduction for a number of reasons, including not being adequate to meet user cooking needs, cost, lack of infrastructure to supply cleaner fuels required, and, in the case of biogas, the prerequisite for parcels of land and animals, to enable use at scale [16]. However, the WHO IAQ Guidelines have highlighted the limitations of ICS in that they cannot sufficiently reduce HAP to levels low enough to prevent ill health. Nevertheless, they do recommend ICS as a transition option from traditional stoves to clean fuels [12]. There are currently hundreds of biomass stoves available to choose from, each with different characteristics, levels of performance, efficiency, indoor emissions, affordability, safety considerations, ability to meet household cooking needs, and importation and shipping costs depending on location. It is therefore a complex question to identify which stove or stoves might be most suitable for specific communities, taking all these aspects into account. This raises an important question for those working in the field; which stove or stoves are most effective at reducing HAP, and are likely to be acceptable and affordable to the global poorest in specific geographical settings?

### 1.3. The World Health Organisation Guidelines on Household Fuel Combustion

**Figure 1:**
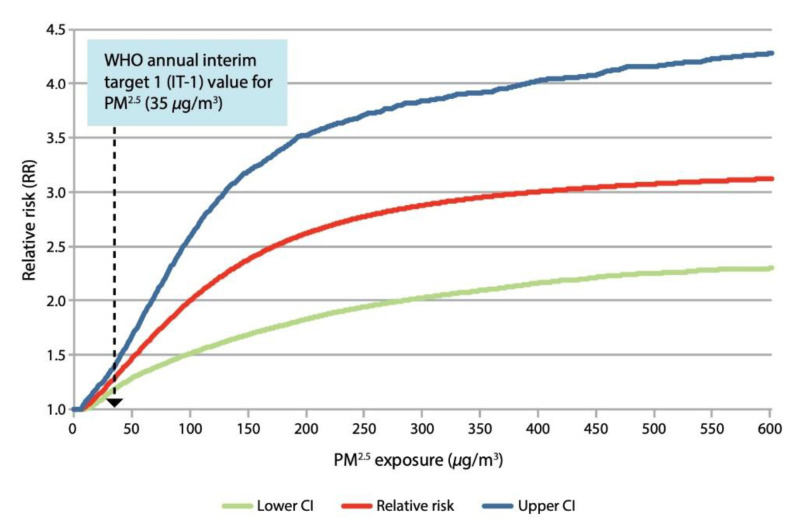
Relationship between the level of PM_2.5_ exposure (µg/m^3^) and the relative risk (95% CI) of child ALRI, based on the integrated exposure–response function, for exposure over the range 0–600 µg/m^3^. Source: WHO (2014) WHO Guidelines for Indoor Air Quality. Geneva. Household Fuel Combustion. p. 43. https://apps.who.int/iris/handle/10665/141496 (last accessed on 30 August 2021) CC BY-NC-SA 3.0 IGO.

The WHO Guidelines provide both an annual target of 10 µg/m^3^ for PM_2.5_ and an interim target (35 µg/m^3^) that stoves and other devices need to meet, in order to reduce childhood acute lower respiratory infections (ALRI) [12] (Figure 1). The targets are based on exposure–response evidence from studies of child ALRI, which demonstrate that due to the non-linear shape of the curve, exposure has to be reduced to ‘at or below 35 μg/m^3^’ to prevent most cases of disease attributable to HAP exposure.

In tandem with this recommendation, the guidelines also demonstrate that even where improved cookstoves have been tested, they are almost exclusively incapable of reducing emissions to a level even close to the interim target. Estimates based on observed concentrations in kitchen field studies published at the time, suggested that approximately 60% of traditional stoves had PM_2.5_ concentrations in the range of 500–1800 µg/m^3^, with a mode of 800 µg/m^3^, and that 60% of improved stoves still had concentration levels in the range of 200–1500 µg/m^3^, with a mode of 500 µg/m^3^, highlighting the inability of the ICS to achieve WHO targets for PM_2.5_ and CO levels able to benefit human health. Despite this limitation, most ICS have been shown to be better alternatives to the traditional stoves or three stone fires in terms of emissions for communities without access to cleaner fuel. Therefore, given that the transition to cleaner fuels will take time, and access to the more expensive technologies available is not feasible to these poorer communities in the near future, the ICS would be an appropriate intermediate step.

The WHO Guidelines led to a greater awareness of the huge variation in emissions from different types of ICS. There are a number of different types of ICS designed to assist better combustion and heat transfer, thereby improving emissions and efficiency performance. The most common types are rocket stoves, a direct combustion-type stove, and gasifier stoves where combustion takes place in two stages. Stoves may also be built with or without a chimney. The chimney stoves tend to be slower, and consume more fuel, but they are generally more effective than non-chimney stoves at reducing household emissions [17]. These different types of stoves demonstrate differing levels of performance, which can be measured using voluntary performance targets. Published in 2018, by the “International Organisation for Standardisation” (ISO) Technical Committee comprised of experts from 45 countries and 8 liaison organizations, these ISO guidelines provide an easily understood ranking of stove performance to allow comparison between different types of stoves [18]. The ISO guidelines allow stoves to be ranked into five tiers based on five different areas of performance: thermal efficiency, CO emissions, PM_2.5_ emissions, safety, and durability, with fine particulate matter being the most important in relation to measuring the health impact in terms of reduced emissions. The emission rates for Tier 5 stoves, the cleanest available, are designed to align with the interim target of WHO Indoor Air Quality Guidelines for PM_2.5_ (35 µg/m^3^). These ISO guidelines allow an assessment to be made, regarding which biomass stove(s) offer the best performance, and can be combined with data available on cost and availability, in order to assess performance alongside availability by geographical region, and also affordability to the poorest communities [19].

## 2. Materials and Methods

The Clean Cooking Alliance Clean Cooking Catalog [19] was utilised as the main data source for this paper. The Catalog includes 500 stoves and 775 separate tests on emissions, efficiency, indoor emissions, and safety based on the ISO standards, and incorporates the best evidence currently available in relation to improved cookstoves. The ISO standards are internationally recognised laboratory performance targets for cookstove testing performance and include thermal efficiency, CO emissions, PM_2.5_ emissions, safety, and durability. To our knowledge, this is the only internationally accepted standard for monitoring the ability of an ICS to reduce HAP. The Clean Cooking Catalog also provides additional information on the type of fuel burnt in each stove, the suggested retail price, the materials used to make the stove, the country in which it is mostly widely used, and market availability. There is, however, no data on user satisfaction.

We searched the database for availability of biomass stoves by cost, market availability, and tier of performance, with an emphasis on indoor emissions (includes a measure of PM_2.5_ and CO) and efficiency, to identify which stoves might be available and accessible to the poorest communities.

As the Clean Cooking Catalog is based on laboratory testing of cookstoves only, we subsequently reviewed the available data (published since the WHO Guidelines release in 2014) from systematic reviews on stove performance in the field. We also searched the literature for any additional factors (such as behavioural or structural actions) reported in field study interventions that could be used to further reduce HAP alongside the use of a cleaner stove. We reviewed this evidence as part of a wider scoping review of field evidence on cookstove interventions, and more information on the methodology of the main scoping review is available via the Open Science Framework [20]. For each paper that was included in the main scoping review, we identified any additional measures that were used in the field to reduce household air pollution, in conjunction with the use of an ICS. We supplemented these sources with findings from all relevant systematic review findings published since 2014 identified through the scoping review methodology.

## 3. Results

### 3.1. Analysis of the Clean Cookstove Catalog

The Clean Cookstove Catalog lists 307 biomass stoves currently on the market. However, only 17 of these stoves are suitable for household use and show evidence of at least Tier 1 reductions in emissions (Table 1). Six stoves show Tier 1 reductions for indoor emissions, six show Tier 2 reductions, three show Tier 3 reductions, and two show Tier 4 reductions.

Considering those stoves which are most effective at reducing HAP, there are no Tier 5 biomass stoves listed, and only two Tier 4 stoves suitable for household use. Of these two stoves, one uses pellets and one stove uses untreated biomass, the Malena (Tier 2 for efficiency and Tier 4 for indoor emissions). The Malena is a chimney stove developed with the Latin American market in mind, so significant work would be needed to explore whether this stove could be adapted and/or produced cheaply enough for an African and Asian market to merit its implementation at scale. Even then, without significant national or international investment, it would need to be built at considerably less cost than the current price of 50 to 70 dollars to fall within the price range of the poorest communities globally. The wood pellet stove is the Mimi Moto manufactured in China, (Tier 4 for efficiency and Tier 4 for indoor emissions) at US$40–65. However, establishing manufacturing and distribution of biomass pellets at scale in Africa would be a costly and complex undertaking [21], and even assuming it could be achieved in a relatively short time period, these stoves are highly unlikely to be affordable to the poorest communities, many of whom do not currently purchase the biomass that they use, making the sustained use of a wood pellet stove even less economically viable [21].

In terms of price, only one of these stoves is priced at under US$5, the Canarumwe, with two additional stoves being available for less than US$20. However, one of these is pellet or woodchips only (THX F11) and one is pellet and wood (THX 14), and as previously, woodchips and pellets are unlikely to be available or affordable to the poorest communities.

It is possible that there are other stoves for less than 20 dollars that could also improve efficiency and indoor emissions, but as yet do not have tier ratings; however, evidence of HAP reduction would be needed if they were to be promoted at scale. The remaining stoves range from between US$25 and US$70, although this does not take into account export and shipping costs, which can be substantial. For the global poorest, all of these stoves would almost certainly not be affordable, given the household daily income of only US$3.20 a day.

Thus, for the poorest communities, only one stove under five dollars can be identified that has undergone IWA tier testing and results in reduced HAP, and even then, the reduction is relatively small, reaching only Tier 1 for indoor emission reductions. Given that there is also the need to potentially consider additional factors, such as importation and shipping costs, and whether a stove meets community cooking needs for example, it is clear that there is a major gap between the SDG7 targets to reduce HAAP among the world’s poorest communities and the affordability and availability of suitable effective interventions to support efforts in the field. As a result, stoves that are shown to be ineffective in reducing indoor emissions are being promoted across low-income countries. In some cases, stoves with recognised improved fuel efficiency, but no reduction in indoor emissions, have been promoted: the Gyapa in Ghana (Tier 2 efficiency and Tier 0 indoor emissions) and the Chitetezo deployed in Kenya, Malawi Mozambique, Rwanda, Zambia, and Zimbabwe (Tier 2 efficiency and Tier 0 indoor emissions), for example. Other promoted stoves, such as the Upesi portable in Kenya (Tier 0 for efficiency and Tier 0 for indoor emissions), the Makaa in Uganda and Kenya (Tier 0 for efficiency and Tier 0 for indoor emissions), and the unrated Mirt stoves disseminated in Ethiopia, show neither a reduction in fuel efficiency nor indoor emissions.

### 3.2. Systematic Reviews Measuring PM_2.5_ Reduction and Health Outcomes from the Use of Improved Cookstoves, Published since the Publication of the WHO IAQ Guidelines

A further important factor to consider in addition to the IWA tier ratings, is the performance of the stoves in the field. Since the publication of the WHO Guidelines, a number of systematic reviews have been carried out in different geographical settings assessing various designs of ICS in relation to health outcomes and indoor emissions reduction. Unlike laboratory measures of stove performance, these studies offer estimates of real-world cooking performance, which differ considerably from performance in the laboratory [22]. These estimates are an important part of assessing the extent to which ICS will reduce HAP in practice. As part of the wider scoping review protocol [20], we identified six relevant systematic reviews that had been undertaken since 2014 when the WHO IAQ Guidelines were published. These reviews focused either on whether ICS are effective in reducing the average concentrations of, or exposure to, particulate matter, or on health outcomes. The reviews include more widely used, relatively high-emission rocket and ceramic Jiko stoves as well as more recently developed low-emission advanced combustion stoves, such as forced draft or semi-gasifier stoves. Some of the reviews provide measures of HAP for specific stoves and others provide measures by stove type. The stoves may or may not include a flue and chimney or smoke hood for ventilation. Table 2 summarises the main findings of systematic reviews that have been carried out since 2014 in relation to HAP estimates from biomass stoves and health outcomes. 

Thus, although there is evidence of a reduction in PM_2.5_ with the use of ICS, none of the systematic reviews report reductions in HAP levels even close to the WHO Guidelines. Further, several of the stoves identified in the studies are now no longer available on the market or are not listed in the Clean Cooking Catalogue and we found no studies included within the systematic reviews, which reported field tests for the Canarumwe stove. However, overall, evidence from field testing does suggest that even modest improvements in indoor air quality have the potential to translate into short-term and potentially long-term improvements in health.

Finally, we also sought to identify any additional factors that could be addressed to contribute to HAP reduction. The factors identified from reviewing the literature and our wider scoping review [20] are listed in Box 1. Given the marginal difference to HAP made by ICS, and recognising that even small improvements in HAP can translate into improvements in health, we would argue that introducing a standard practice in the field of always focusing on additional practices, rather than an exclusive focus on the ICS, would enable a suite of different practices to be considered by communities and could result in further reductions in HAP for communities. Such a list of interventions would need to be reviewed and adapted by local communities to ensure it addressed the potential for actions that are appropriate to the setting in which the ICS are being used.

Box 1 **Potential interventions to consider alongside cleaner cookstoves.**

Drying wood before burning

Burning rubbish away from the household

Removing children from the vicinity of the ICS

Smoking reduction approaches

Use of wonderbags (non-electric heat-retention cookers)

Training to avoid over-stuffing of ICS

Solar and battery lighting to replace kerosene

Use of pot lids to reduce cooking times

Consideration of improved ventilation

Cooking outside

Alternative practices to burning agricultural waste

Community centred participatory approaches

Increased use of lay knowledge in stove uptake and implementation.

## 4. Discussion

In order for policy makers and programme implementers to make informed decisions about which ICS to promote, clear guidance is required about the benefits and limitations of the different ICS, especially in relation to their ability to reduce emissions. Whilst there are data on ISO standards for stoves, this information is not available for many stoves on the market, and further, many of the stoves for which it is available have only been tested under laboratory conditions and not in the field.

Large numbers of so-called improved stoves are still being disseminated, for which there is no evidence of effectiveness in reducing indoor emissions. It is difficult to justify promoting these unrated stoves. However, for some ICS, there is evidence that their fuel- and time-saving benefits due to increased efficiency, do improve quality of life for the user, regardless of whether there are discernible health benefits during lab testing [29]. In particular, where households pay for wood, a reduction in fuel use may offer substantial money saving, so the argument for distributing more fuel-efficient stoves may be a valid one, particularly if it is combined with additional practices to reduce HAP. However, it would be preferable to disseminate ICS that have reduced emissions as well as improved efficiency.

A single source of information is required that allows identification of the cleanest stoves available within an identified price range that households can afford, which also takes into account the importance of adequately meeting community cooking needs, import and shipping costs, and availability. Cookstoves vary considerably in terms of type and characteristics, including emissions performance, safety, efficiency, in-field performance, sustainability, affordability, and availability in different settings, as well as the ability to meet user cooking needs. Whilst there is a large body of evidence available, it is not in a form that can be used to determine which stove may be most suitable for a specific community. The Global Alliance Clean Cookstove Catalogue [19] is the most comprehensive source currently available and includes data on 500 stoves (325 biomass stoves available on the market), including price and laboratory performance, but it does not provide a comparison of performance in the field, information on user perspectives, or information on importation costs and availability of ICS in different international settings. Further, whether or not improved cookstoves can realistically meet community cooking needs in the field, is a critical factor when it comes to sustained and exclusive use of ICS. Numerous studies exploring cleaner cooking in respect of user and community needs and perceptions have been carried out [29,30], and the findings of these studies and other relevant publications should be assessed alongside information on stove characteristics, laboratory measured indoor emissions, etc., to provide more complete data on which factors the clean cookstove sector can base decisions, regarding stove suitability.

In terms of field study data, these studies are crucial as they offer real-world estimates of different stoves in different settings. Field-test data accompanied by laboratory data, by stove make, for those stoves that are currently available on the market, would be a really useful addition. There is also some evidence that biomass stoves with chimneys perform better than those without [25,31] and adaptation of chimney stoves to meet the needs of the global poorest may be an option worth exploring further.

In addition, intervention studies in real-world settings are required for locally made stoves where there may not yet be laboratory or real-world evidence of HAP reduction. For these stoves, field measurements should always be carried out prior to distribution, and publication of these results in the literature would allow greater opportunity for performance comparison between different locally made stoves, allowing guidelines for building cleaner locally made stoves to be drawn up. A detailed scoping of the literature to map out all these different factors would enable clear identification of which stove might be most appropriate for which context.

Returning to the different sources of HAP in the global poorest communities, there are a number of additional sources of pollution and also community practices that are not being systematically addressed in the cleaner cooking field. Some of these are directly related to the stove itself (such as the burning of wet wood) and others relate to other sources of HAP (and in some cases HAAP), such as kerosene lighting. Some of these factors could be addressed relatively simply (through the provision of wood storage and availability of solar lighting for instance).

In order to realistically reduce HAP in the poorest communities, it is therefore important to widen the focus to incorporate other practices and technologies that can contribute to reducing the impact of HAP on the poorest communities globally, to help them transition to clean and modern energy. In tandem with this approach, a number of researchers have called for a greater focus on the importance of behaviour change approaches [32,33,34,35] alongside the introduction of new technologies, suggesting that emphasis on the technology alone has been a major issue in the success (or lack thereof) of many programmes. According to Sesan et al. [33], the overwhelming focus on distributing stoves in the cookstove sector rather than on why households would want to use a cookstove or prioritise a smoke-free household is a significant potential barrier to success. Essentially, communities are unlikely to change their practices when clean cooking ranks well below other priorities or needs. Sesan et al. also argue that research is required into intrinsic motivators for change in different local contexts, and that these motivators may be used as the starting point for responsive multi-level interventions in the cookstove sector. The Multi–Tier Framework for measuring access to cleaner cooking offers one approach to measuring the household-level impacts of a suite of different approaches, as it measures exposure, efficiency, convenience, safety, and affordability. If convenience is interpreted as the ability of an intervention to meet household cooking needs, this is a useful framework with which to consider different actions and interventions aimed at supporting the move towards cleaner energy [36].

Further, the move away from ‘top–down supply-led’ approaches towards a more participatory approach would increase community engagement and provide a platform for the community voice to be heard, especially in a field where household and community ‘buy in’ is key to the successful uptake and sustainable use of cleaner cooking practices and behaviours. Local communities provide valuable insights into the barriers and facilitators they face in relation to the use of cleaner cooking and lighting practices. In a previous paper, we recommended giving more weight to lay knowledge, and in particular, directly seeking to address the issues that users face in relation to their everyday cooking practices, to enable their views to more effectively inform practices that will reduce HAP exposure [37]. This knowledge needs to be integrated with the stove factors discussed above, so that informed decisions can be made about which stoves and stove practices are likely to be appropriate for and acceptable in different community settings.

Finally, this paper has not discussed the wider issues that are relevant to national scale up, but has focused on what can be implemented at a user and community level. Clearly, there are other policy factors that need to be considered beyond the community setting to address broader issues that act as barriers to clean cooking, such as providing micro-finance or introducing carbon credits, for example, which could be of significant benefit in the community setting. National and regional policy that supports communities to reduce HAP is an extremely important aspect of enabling a community to be successful in their approaches to reducing HAP. Any national or international transition to greater access to clean energy cannot be carried out at the community level without national political will, and strong coalitions, in order to meet the aspirations of SDG 7.1. [36].

## 5. Conclusions

Access to modern energy for all (SDG 7) is crucial to the success of most of the other 16 SDGs. Despite energy playing a critical role in economic development and health, the world remains severely off track in relation to achieving universal access to affordable, reliable, sustainable, and modern energy for all by 2030 [38]. In terms of reducing HAP in low-income countries, identifying the most appropriate cooking intervention(s), integrating lay knowledge of stove suitability, and adopting a more holistic approach that includes additional interventions to reduce exposure, offer a plausible way forward to reducing HAAP in the poorest communities worldwide.

Given that poorer communities will not have access to gas, electricity, or the more expensive technologies currently under development for many years to come, and recognizing that intermediate steps are required, we therefore recommend the following:

- Stove developers should be cognizant of the need to develop stoves that will be affordable to the poorest communities. Research funding focusing on producing affordable stoves that reduce HAP would improve the current availability of stoves to the poorest communities globally.
- ICS with HAP benefits should be promoted where possible. Where no stove is available which reduces HAP, the promotion of stoves that increase efficiency and reduce fuel use could be considered an intermediate step, although it must be recognized that they offer no direct health benefits to the user.
- Clear guidance is required to enable the identification of the most appropriate cookstove to promote to a community. This guidance should take into account the type and characteristics of the stove, stove sustainability, safety, emissions performance, efficiency, in-field performance, affordability, availability in different settings, and also the ability of the stove to meet community cooking needs. Further, it is important for field studies to outline the stove brand, model, and price to aid in knowledge translation and stove recommendations. A detailed scoping review is required to bring all of this information together in a format that is accessible for the clean cooking sector. A toolkit that supports community and programme leads in making local decisions by taking into account affordability, accessibility, acceptability, and sustainability issues alongside relevant national and legal policy would enable a more systematic approach to be taken to the introduction of HAP-reducing measures.
- Currently, of the 343 biomass ICS listed in the Clean Cookstove Catalog and available on the market, 185 (almost 60%) do not show evidence of any testing at all, either via the IWA tiers or by individual testing centres. Undergoing IWA tier rating testing and performance testing in the field should be a requirement for any ICS being promoted in the field.
- A suite of interventions should be promoted alongside cleaner stoves, including cleaner lighting, alternatives to burning crops and rubbish, drying of wood and adequate wood storage, smoking reduction, improved ventilation, outdoor cooking, and behavioural interventions, such as smoking cessation, reduced time spent close to the fire, use of pot lids and wonderbags to reduce cooking times, and burning of rubbish away from households. These options can be presented to communities to enable them to identify feasible interventions to implement in their own settings.
- The Malena offers a relatively cheap cookstove with Tier 4 benefits in relation to HAP reduction. The question remains as to whether an adapted Malena design, built to meet the cooking needs of households in the poorest communities in Africa and Asia, might offer a feasible alternative given that chimney stoves show promise in reducing HAP. This merits greater exploration for the poorest communities in Africa and Asia as an interim measure over the next twenty years.
- The cookstove sector should actively work with local communities at the planning stage of any intervention, in order to introduce approaches that are tailored to the community and take their views into account. Issues of affordability, accessibility, sustainability, and acceptability are crucial and point to the importance of focusing on the needs and perspectives of the user. Local communities provide valuable insights and are well positioned to identify enablers and barriers in relation to certain practices and contribute to identifying solutions. They should therefore be involved throughout all stages of programme or project development and implementation aimed at reducing HAAP.

## Figures and Tables

**Table 1:** Household biomass stoves available on the market (Tier 1 and above for indoor emissions).

Name of Stove	Cost (US$)	Stove Characteristics	Country of Manufacture	Indoor Emissions (IWA Tier)	Efficiency (IWA Tier)
**Tier 1**					
Canarumwe	3–4	Household, Built in place, Ceramic-lined, Traditional	Rwanda	1	1
Berkeley Darfur V.14	25–35	Non-traditional, Pot skirt, Sunken pot, Multiple burners, Rocket, Portable, Household	India	1	2
Ezystove	25–75	Household, Rocket, Non-traditional, Portable, Side-feed	China	1	1
Augusta	40 to 45	Side-feed, Non-traditional, Household, Portable, Rocket	Bolivia	1	2
Prime square granular regular	25–45	Non-traditional, Portable, Gasifier (TLUD), Household, Batch loaded	Indonesia,	1	2
Smartsaver wood	N/A	Household, Portable, Non-traditional	Information Not available	1	2
**Tier 2**					
THX14 (pellets and wood)	5–12	Household, Portable, Batch loaded, Gasifier (TLUD)	Vietnam	2	0
THX F11 (pellets and woodchips)	18–22	Household, Portable, Batch loaded, Gasifier (TLUD), Fan, Pot skirt	Vietnam	2	0
Prime square F’wood regular	25–45	Portable, Batch loaded, Gasifier (TLUD), Non-traditional, Household	Indonesia,	2	2
Biolite home stove	40–70	Fan, Thermoelectric generator, Non-traditional, Household, Portable, Side-feed, Pot skirt	China	2	2
Prime square fuelwood regular	25–45	Portable, Batch loaded, Gasifier (TLUD), Non-traditional, Household	Indonesia	2	2
Kuniokoa	N/A	Non-traditional, Household, Portable, Side-feed, Rocket	Kenya	2	2
**Tier 3**					
Ace 1	N/A	Non-traditional, Household, Portable, Gasifier (TLUD), Ceramic-lined, Fan	Lesotho	3	3
GAMA1411	N/A	Traditional, Portable, Heating, Chimney, Non-traditional, Household, Side-feed	Bolivia and Peru	3	2
Oorja (pellets)	N/A	Batch loaded, Gasifier (TLUD), Non-traditional, Portable, Fan, Ceramic-lined	India	3	2
**Tier 4**					
Malena	50–70	Non-traditional, Household, Built in place, Rocket, Chimney, Sunken pot, Side-feed	Bolivia	4	2
Mimi moto	40–65	Non-traditional, Household, Portable, Batch loaded, Gasifier (TLUD), Fan, Solar: Panel	China	4	4

Data obtained from the Clean Cooking Catalog Available online: http://catalog.cleancookstoves.org/stoves (accessed on 22 July 2021). N/A = not available.

**Table 2:** Systematic Reviews Measuring PM_2.5_ Reduction and Health Outcomes from the Use of Improved Cookstoves, that have been published since the Publication of the WHO IAQ Guidelines.

Authors	No. of Included Studies	HAP Estimates and Health Outcomes
Thomas et al. 2015 [23]	36—variety of study designs, including 11 RCT’s	The majority of studies produced a positive effect on HAP levels with an improved cookstove. Meta-analysis not feasible due to different measurements used.
Thakur et al. 2017 [24]	53—variety of study designs, including 21 RCT’s	No impact on paediatric lower respiratory tract infections, severe pneumonia, miscarriage, stillbirth or infant mortality. Significant reduction (self) reported for cough, wheezing, breathing difficulties and conjunctivitis.
Pope et al. 2017 [25]	42 studies included (no of RCT’s not reported in main paper). Some studies included use of cleaner fuels as well as biomass.	Large reductions in pooled kitchen PM_2.5_ of 41% (29–50%) for advanced combustion stoves. Biomass stoves with chimneys performed better than those without. However, post-intervention kitchen PM_2.5_ levels remained well above WHO IAQ recommended limits.
Quansah et al. 2017 [26]	55—variety of study designs, including 11 RCTS	PM_2.5_ was reduced by up to 67% but this differed by stove type. Levels however were still significantly above WHO IAQ guidelines Current ‘stand-alone’ HAP interventions yield little benefit. Findings on health outcomes inconclusive.
Onakomaiya et al. 2019 [27]	5 studies Including 3 RCTs	Limited findings but some evidence that effects on blood pressure are significant demonstrating a lowering in systolic and diastolic blood pressure.
Saleh et al. [28]	14 studies, 12 testing improved cookstoves—all included studies were RCTs	No studies demonstrated a significant benefit in child pneumonia outcomes. Improvements seen with reported respiratory symptom outcomes with some, but self-reporting made these outcomes vulnerable to bias.

## Data Availability

Data used are publicly available.
